# Botulinum toxin A complex exploits intestinal M cells to enter the host and exert neurotoxicity

**DOI:** 10.1038/ncomms7255

**Published:** 2015-02-17

**Authors:** Takuhiro Matsumura, Yo Sugawara, Masahiro Yutani, Sho Amatsu, Hideo Yagita, Tomoko Kohda, Shin-Ichi Fukuoka, Yutaka Nakamura, Shinji Fukuda, Koji Hase, Hiroshi Ohno, Yukako Fujinaga

**Affiliations:** 1 Laboratory for Infection Cell Biology, International Research Center for Infectious Diseases, Research Institute for Microbial Diseases, Osaka University, 3-1, Yamada-oka, Suita, Osaka 565-0871, Japan; 2 Department of Immunology, Juntendo University School of Medicine, 2-1-1 Hongo, Tokyo 113-8421, Japan; 3 Department of Veterinary Sciences, School of Life and Environmental Science, Osaka Prefecture University, 1-58, Rinku-oraikita, Izumisano, Osaka 598-8531, Japan; 4 School of Culture and Creative Studies, Aoyama Gakuin University, Tokyo 150-8366, Japan; 5 Department of Biochemistry, Faculty of Pharmacy, Keio University, Tokyo 105-8512, Japan; 6 Laboratory for Intestinal Ecosystem, RIKEN Center for Integrative Medial Sciences (IMS), Yokohama, Kanagawa 230-0045, Japan; 7 Department of Medical Life Science, Graduate School of Medical Life Science, Yokohama City University, Yokohama, Kanagawa 230-0045, Japan; 8 Department of Immune Regulation, Graduate School of Medicine, Chiba University, Chiba 260-8670, Japan

## Abstract

To cause food-borne botulism, botulinum neurotoxin (BoNT) in the gastrointestinal lumen must traverse the intestinal epithelial barrier. However, the mechanism by which BoNT crosses the intestinal epithelial barrier remains unclear. BoNTs are produced along with one or more non-toxic components, with which they form progenitor toxin complexes (PTCs). Here we show that serotype A1 L-PTC, which has high oral toxicity and makes the predominant contribution to causing illness, breaches the intestinal epithelial barrier from microfold (M) cells via an interaction between haemagglutinin (HA), one of the non-toxic components, and glycoprotein 2 (GP2). HA strongly binds to GP2 expressed on M cells, which do not have thick mucus layers. Susceptibility to orally administered L-PTC is dramatically reduced in M-cell-depleted mice and GP2-deficient (*Gp2*^−/−^) mice. Our finding provides the basis for the development of novel antitoxin therapeutics and delivery systems for oral biologics.

## Abstract (web summary)

It is unclear how ingested botulinum neurotoxin invades the host to cause illness. Here, the authors show that the toxin complex containing neurotoxin, hemagglutinin (HA), and NTNHA proteins traverses the epithelial barrier via HA-glycoprotein 2 interaction and endocytosis by Peyer’s patch microfold cells.

## Graphical abstract

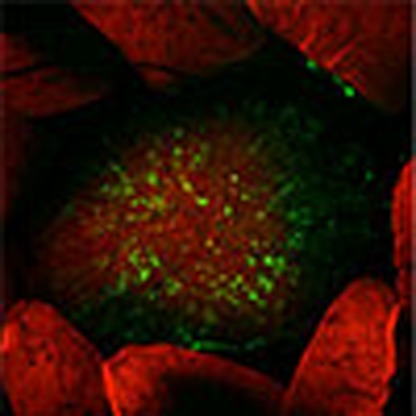

Botulinum neurotoxin (BoNT), which is produced by *Clostridium botulinum* and related species, is a potent metalloprotease toxin consisting of a large protein (~150 kDa) that binds neuronal cells^1^. On entering the cytoplasm of these cells, it cleaves SNAREs (soluble *N*-ethylmaleimide-sensitive fusion protein attachment protein receptors) required for vesicle fusion, thereby inhibiting neurotransmitter release and causing paralysis^1^. BoNTs are produced along with one or more neurotoxin-associated proteins (NAPs) that non-covalently associate with BoNT to form progenitor toxin complexes (PTCs)^2^ (Fig. 1a). The NAPs include non-toxic non-hemagglutinin (NTNHA) and hemagglutinin (HA). HA is composed of three different subcomponents: HA1, HA2 and HA3 (alternatively, HA-33, HA-17 and HA-70, respectively)^3^. HA1 and HA3 have carbohydrate-binding activities^4^. *C. botulinum* type A1 strains produce M-PTC, L-PTC and LL-PTC simultaneously^2^. M-PTC contains BoNT and NTNHA^5^, whereas L-PTC consists of BoNT, NTNHA and HA^6^,^7^. LL-PTC is assumed to be a dimer of L-PTC^8^, and dilution of concentrated LL-PTC leads to dissociation into L-PTC^9^. Ingestion of foods contaminated with PTCs causes food-borne botulism, the most common form of botulism in adults^10^. The presence of NAPs in PTCs drastically increases BoNT toxicity following oral administration^2^. At least three mechanisms possibly involved in this phenomenon have been reported: protection of BoNT by NTNHA and HA against degradation in the gastrointestinal tract^2^,^11^; promotion of binding to intestinal epithelial cells through the carbohydrate-binding activity of HA^12^ and disruption of the epithelial barrier via an interaction between HA and E-cadherin^13^,^14^,^15^,^16^.

Intestinal absorption of BoNT is essential for the onset of food-borne botulism. However, the invasion site(s) and mechanism of BoNT *in vivo* are largely unknown. Here we analyze the site(s) responsible for intestinal translocation of the type A1 BoNT (BoNT/A1) complex *in vivo* and molecular mechanisms involved in this step. L-PTC, which makes the predominant contribution to causing illness, binds to microfold (M) cells in the follicle-associated epithelium (FAE) of mouse Peyer’s patches (PPs), and is transported to their basolateral sides via the interaction of HA in the L-PTC with glycoprotein 2 (GP2) on the M-cell surface. Susceptibility to orally administered L-PTC is dramatically reduced in M-cell-depleted mice and GP2-deficient (*Gp2*^−/−^) mice. Our data reveal that to exert its toxic effects, type A1 L-PTC invades the intestinal epithelium through M cells via HA–GP2 interaction.

## Results

### L-PTC is taken up by M cells via HA

**Figure 1:**
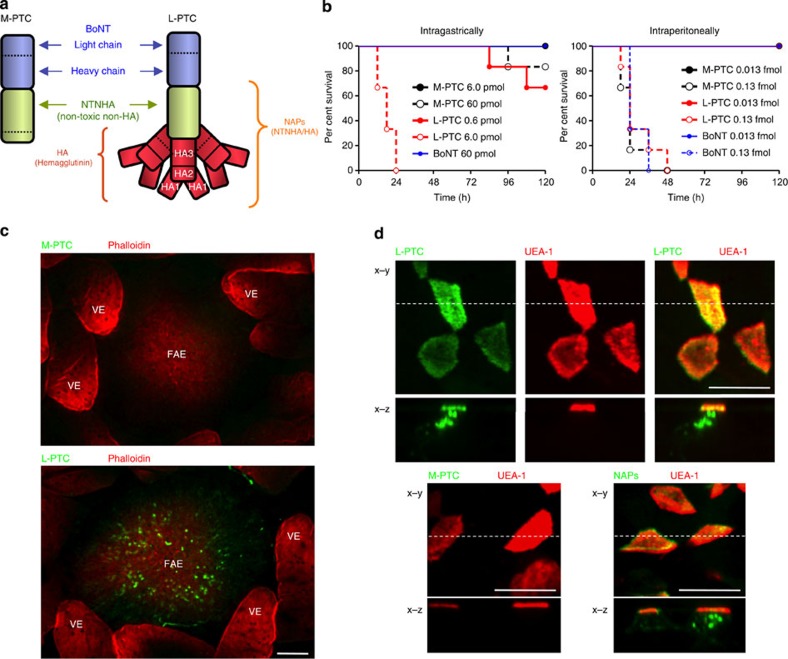
L-PTC is taken up by Peyer’s patch M cells. (**a**) Schematic representation of botulinum neurotoxin complexes. (**b**) Various concentrations of toxins were intragastrically (M-PTC 6.0 pmol: 1.72 μg, 60 pmol: 17.2 μg, L-PTC 0.6 pmol: 0.45 μg, 6 pmol: 4.5 μg, BoNT 60 pmol: 9.0 μg) or intraperitoneally (M-PTC 0.013 fmol: 3.85 pg, 0.13 fmol: 38.5 pg, L-PTC 0.013 fmol: 10 pg, 0.13 fmol: 100 pg, BoNT 0.013 fmol: 2.01 pg, 0.13 fmol: 20.1 pg) administered to mice (*n*=6 per group). (**c**) Alexa Fluor 488-labelled toxins (green) were injected into ligated mouse intestinal loops containing PPs, and incubated for 2 h. Whole-mount intestinal villous epithelium (VE) regions and FAE regions were stained with Alexa Fluor 568-labelled phalloidin (red). (**d**) Alexa Fluor 488-labelled toxins or NAPs (NTNHA/HA, green) were injected into ligated mouse intestinal loops; FAE regions were stained with rhodamine-labelled UEA-1 (red). *x*–*z* images in lower panels correspond to the positions indicated by dotted lines in the *x*–*y* images. Scale bars, 100 μm (**c**), 10 μm (**d**). The data in **c**,**d** are representative of three independent experiments.

To compare the toxicities of BoNT/A1 alone, M-PTC and L-PTC, we intragastrically or intraperitoneally administered each toxin to mice (Fig. 1b). When intragastrically administered, the toxicity of L-PTC was highest, ~2 orders of magnitude greater than that of M-PTC and M-PTC was more toxic than BoNT alone. By contrast, there was no significant difference among the toxicities of the intraperitoneally administered toxins. These results indicate that L-PTC makes the predominant contribution to the onset of food-borne botulism.

To determine the site(s) responsible for intestinal translocation of the toxin, we assessed localization of PTCs by *in situ* intestinal loop assays in mouse. L-PTC was selectively localized at the FAE that covering PPs, whereas M-PTC exhibited no such clear localization to any sites in the intestinal tissue (Fig. 1c). These data imply that L-PTC binds to, and is internalized by, specific cells present in the FAE. Therefore, we focused on the M cells, which are present in the FAE. These cells effectively bind and deliver luminal macromolecules to the cells of underlying mucosal immune system for the induction of intestinal immune responses^17^. However, M-cell-dependent antigen uptake process can be exploited by some pathogens^18^. Indeed, L-PTC, its NAPs (a complex of NTNHA/HA) and HA bound to *Ulex europaeus* lectin 1 (UEA-1)+ M cells, and were then transported to their basolateral sides (Fig. 1d and Fig. 5b). By contrast, M-PTC exhibited minimal interaction with M cells. Thus, HA is the critical factor in the interaction with M cells. After a 3-h incubation, L-PTC was located on the basolateral side of the FAE and in CD11c+ dendritic cells (CD11c+ DCs), which are located in the sub-epithelial dome (Supplementary Fig. 1a). Using L-PTC reconstituted with Alexa Fluor 488-labelled BoNT and Alexa Fluor 568-labelled NAPs, we also observed that CD11c+ DCs, localized beneath the M cells harbouring L-PTC and captured both BoNT and NAPs, which were only partially co-localized (Supplementary Fig. 1b,c, Supplementary Movie 1). Most of the NAPs were dissociated from L-PTCs and located predominantly in M cells, whereas most of the BoNTs were located in CD11c+ DCs. This observation was consistent with the previous proposal that NAPs, which are associated with BoNTs in the luminal environment of the intestine, dissociate after crossing the intestinal epithelium^2^. It remains unclear what percentage of BoNT present in the sub-epithelial dome is trapped by CD11c+ DCs. In any case, these observations indicate that all the constituents of L-PTC, including BoNT and NAPs, were transcytosed across the M cells to the basolateral surface, and captured by CD11c+ DCs. Furthermore, *in vivo*, L-PTCs were taken up by M cells in the small intestine 1 h after oral administration (Supplementary Fig. 2). Collectively, these results suggest that M cells play an important role in the transepithelial delivery of L-PTC.

### M cells are the major sites of L-PTC invasion *in vivo*

**Figure 2:**
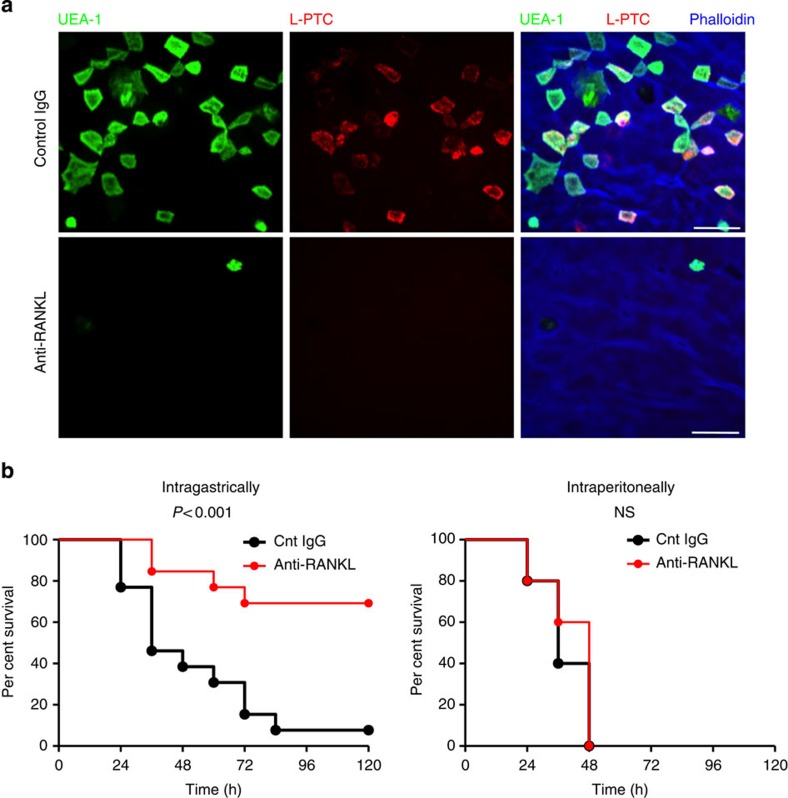
M cells are the major sites at which L-PTC breaches the intestinal epithelial barrier. (**a**,**b**) Mice were treated intraperitoneally with anti-RANKL antibody or control rat IgG. (**a**) Alexa Fluor 568-labelled L-PTC (red) was injected into ligated mouse intestinal loops and incubated for 2 h, and FAE regions were stained with FITC-labelled UEA-1 (green) and iFluor 405-labelled phalloidin (blue). Scale bar, 20 μm. (**b**) L-PTC was intragastrically (1.0 pmol: 0.75 μg) or intraperitoneally (0.067 fmol: 50 pg) administered to mice treated with anti-RANKL antibody or rat IgG (intragastrically, *n*=13 per group; intraperitoneally, *n*=5 per group). Statistical analyses were performed with the log-rank test. NS, not significant. The data (**a**) are representative of two independent experiments.

We next confirmed the biological significance of M cells as a portal for L-PTC *in vivo*. Signalling via the TNF superfamily member receptor activator of NF-κB ligand (RANKL) and receptor activator of NF-κB (RANK) plays a key role in the differentiation of M cells, which can be transiently depleted by RANKL neutralization^19^,^20^. In mice treated with monoclonal antibody against RANKL, the number of M cells was drastically reduced relative to control mice, and this reduction was accompanied by loss of localization of L-PTC at FAE (Fig. 2a). Correspondingly, susceptibility to orally administered L-PTC was significantly reduced in the mice treated with anti-RANKL antibody. By contrast, M-cell depletion did not influence lethality on systemic challenge with L-PTC (Fig. 2b) or on oral administration of M-PTC (Supplementary Fig. 3). These results provide evidence that M cells make a significant contribution as a major portal for L-PTC.

### GP2 serves as a major endocytotic receptor for L-PTC

**Figure 3:**
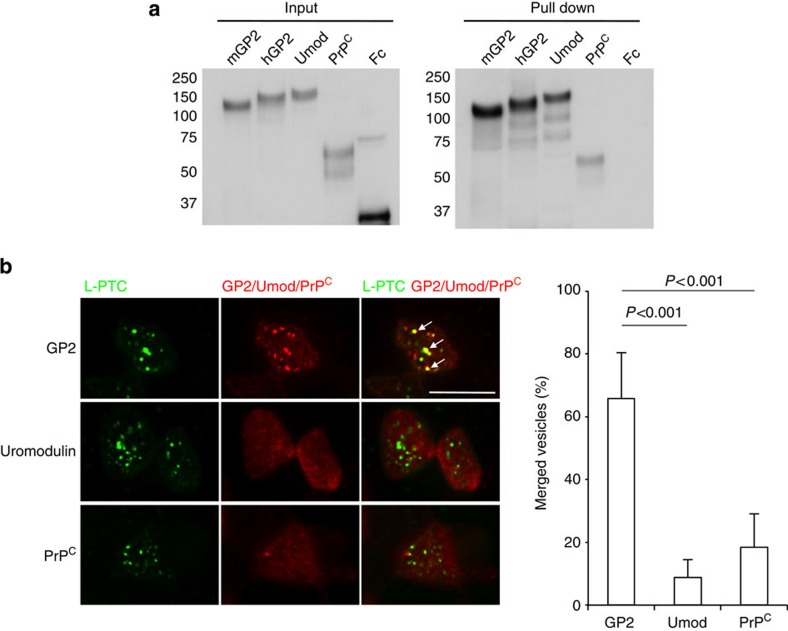
Glycoprotein 2 (GP2) serves as a major endocytotic receptor for L-PTC in M cells. (**a**) Recombinant Fc proteins were incubated with Strep-Tactin Superflow agarose pre-bound to fully assembled HA (HA1/HA2/HA3). HA-bound proteins were analyzed by immunoblotting using HRP-labelled anti-human IgG antibody. (**b**) Alexa Fluor 488-labelled L-PTC (green) were injected into ligated mouse intestinal loops and incubated for 1 h; GP2, uromodulin and PrP^C^ were stained with specific antibodies and Cy3-labelled secondary antibodies (red). L-PTC predominantly co-localized with GP2 in M cells (arrows). For three-dimensional imaging of co-localization of L-PTC with GP2, see Supplementary Movie 2. Co-localization of L-PTC with GP2, uromodulin or PrP^C^ in the randomly selected M cells was counted (*n=*15), and the data are expressed as the number of merged L-PTC vesicles divided by the total number of L-PTC vesicles. Error bars indicate s.d. Statistical analyses were performed with Student’s *t*-test. Scale bar, 10 μm. Data are representative of two (**b**) or three (**a**) independent experiments.

**Figure 4:**
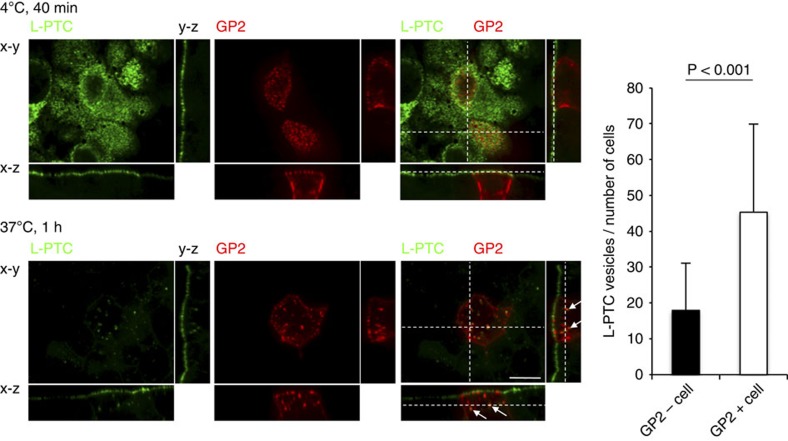
GP2 promotes the endocytotic uptake of L-PTC. MDCK I monolayers were transiently transfected with mGP2, and seeded on transwells. Monolayers were apically treated with L-PTC (200 nM) for 40 min at 4 °C (upper panels) or 1 h at 37 °C (lower panels). L-PTC was labelled with anti-L-PTC antibody and Alexa Fluor 488-labelled anti-rabbit IgG antibody (green), and GP2 was labelled with anti-GP2 antibody and Cy3-labelled anti-rat IgG antibody (red). Endocytotic vesicles of L-PTC in GP2-negative (GP2−) or GP2-positive (GP2+) cells were counted (GP2− cells, 72 cells; GP2+ cells, 35 cells), and the data are expressed as total L-PTC vesicles per number of cells. Error bars indicate s.d. Statistical analyses were performed with Student’s *t*-test. L-PTC co-localized with GP2 in GP2+ cells (arrows). Scale bar, 10 μm. Data are representative of two independent experiments.

It is well-known that efficient uptake by M cells requires specific surface receptor molecules such as glycosylphosphatidylinositol-anchored proteins: for example, GP2 serves as a transcytotic receptor for *Salmonella enterica* serovar Typhimurium and *Escherichia coli*^21^, whereas cellular prion protein (PrP^C^) interacts with *Brucella abortus*^22^. Uromodulin (Umod, also known as Tamm-Horsfall protein; THP), a binding molecule for uropathogenic *E. coli* expressed by renal tubular epithelium^23^, is also expressed on M cells^24^. Therefore, we next asked whether these molecules could serve as receptors for L-PTC. HA strongly bound both mouse and human GP2, to a lesser extent to mouse uromodulin and weakly to mouse PrP^C^ (Fig. 3a). Next, we examined the co-localization of L-PTC and these three molecules in M cells in *in situ* intestinal loop assays (Fig. 3b). Approximately 65% of the L-PTC-harbouring endocytic vesicles co-localized with GP2 (also see Supplementary Movie 2), whereas the L-PTC-harbouring endocytic vesicles co-localized to a lesser extent with uromodulin and PrP^C^ (~10% and 20%, respectively). These data are consistent with the observation that GP2 was expressed in M cells at much higher levels than the other two molecules (Supplementary Fig. 4). When Madin–Darby canine kidney I (MDCK I) monolayers were transiently transfected with mouse GP2, L-PTC strongly bound to the apical surface of MDCK I monolayers regardless of whether GP2 was present (Fig. 4). Of note, however, even in this experimental setting, L-PTC was highly co-internalized with GP2, and endocytosis of L-PTC was significantly elevated in GP2-expressing MDCK I cells relative to cells that did not express GP2 (Fig. 4). Collectively, these data indicate that GP2 serves as a major endocytotic receptor for L-PTC in M cells.

### HA recognizes the sugar chains present on GP2

**Figure 5:**
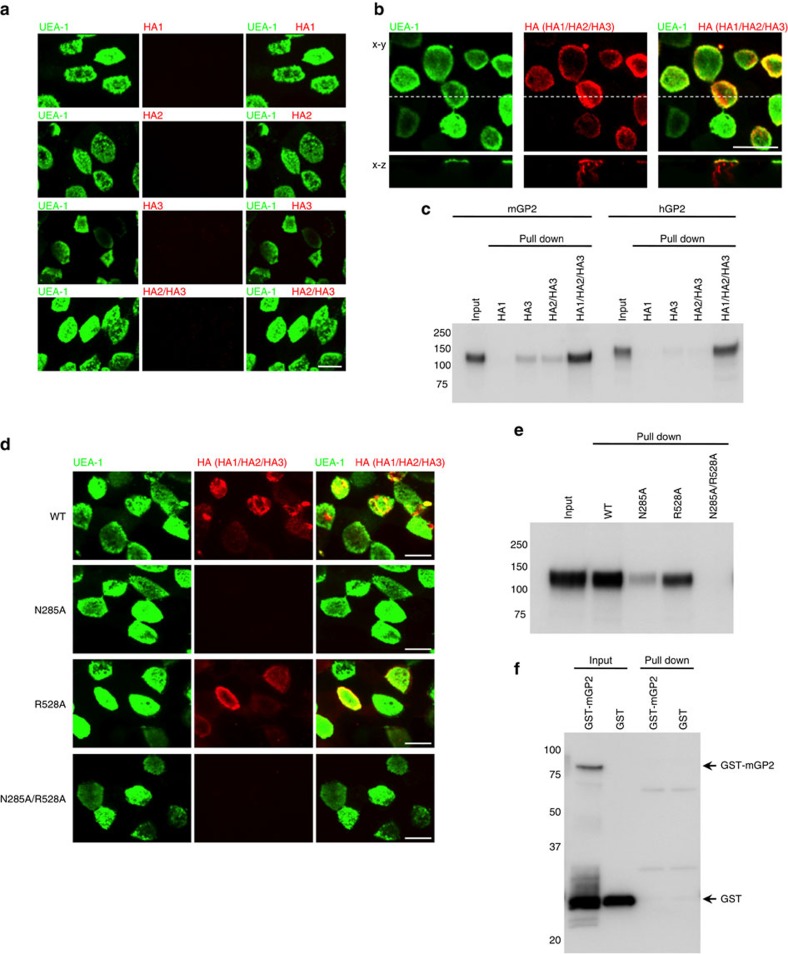
Binding of HA with GP2 is mediated by the carbohydrate-binding activities of HA. (**a**,**b**) Alexa Fluor 568-labelled HA subcomponents or reconstituted HA (Alexa Fluor 568-labelled HA2/HA3 or HA1/Alexa Fluor 568-labelled HA2/HA3; red) were injected into ligated mouse intestinal loops and incubated for 2 h; FAE regions were stained with FITC-labelled UEA-1 (green). (**c**) Recombinant Fc proteins (mGP2 and hGP2) were incubated with Strep-Tactin Superflow agarose pre-bound to HA1, HA3, HA2/HA3 core complex or HA (HA1/HA2/HA3). HA-bound proteins were analyzed by immunoblotting using HRP-labelled anti-human IgG antibody. (**d**) Reconstituted WT HA (HA1/Alexa Fluor 568-labelled HA2/HA3), mutant HA complex harbouring mutant HA1 (N285A, HA1N285A/Alexa Fluor 568-labelled HA2/HA3), mutant HA complex harbouring mutant HA3 (R528A, HA1/Alexa Fluor 568-labelled HA2/HA3R528A) and mutant HA complex harbouring mutant HA1 and HA3 (N285A/R528A, HA1N285A/Alexa Fluor 568-labelled HA2/HA3R528A; red) were injected into ligated mouse intestinal loops and incubated for 2 h; FAE regions were stained with FITC-labelled UEA-1 (green). (**e**) Recombinant mGP2-Fc protein was incubated with Strep-Tactin Superflow agarose pre-bound to HA (WT, N285A, R528A or N285A/R528A). HA-bound proteins were analyzed by immunoblotting using HRP-labelled anti-human IgG antibody. (**f**) GST–mGP2 and GST were incubated with Strep-Tactin Superflow agarose pre-bound to HA (WT). HA-bound proteins were analyzed by immunoblotting using anti-GST antibody and HRP-labelled anti-rabbit IgG antibody. Scale bar, 10 μm (**a**,**b**,**d**). Data are representative of two (**a**,**d**) or three (**b**,**c**,**e**,**f**) independent experiments.

Next, we attempted to identify the critical subcomponent of HA required for the interaction with GP2 and localization of L-PTC to M cells. Individual HA subcomponents and the HA2/HA3 core complex formed by HA2 and HA3 (ref. 3) exhibited little or no binding to GP2 and no localization to M cells. By contrast, whole HA reconstituted with all three subcomponents strongly bound GP2 and localized to M cells in a manner similar to L-PTC (Fig. 5a–c). These results indicate that integration of all the HA subcomponents is required for the interaction with GP2, as well as for the localization of L-PTC to M cells. The fact that fully assembled HA is required for the interaction with GP2, even though the single subcomponents of HA1 and HA3 exhibit carbohydrate-binding activities^4^, suggests that these carbohydrate-binding activities are insufficient, either because single HA subcomponents lack sufficient avidity for robust binding or because the isolated subcomponents lack a three-dimensional protein structure formed by the HA1/HA2/HA3 assembly involved in recognition of GP2. On the other hand, the presence of galactose inhibited binding between L-PTC and GP2 (Supplementary Fig. 5a), and L-PTC bound deglycosylated GP2 less strongly than intact GP2 (Supplementary Fig. 5b). To confirm the role of the carbohydrate-binding activities in GP2 recognition, we generated mutant HA complexes with an inactivated galactose-binding site (HA1:N285A/HA2/HA3, designated as N285A), an inactivated sialic acid binding site (HA1/HA2/HA3:R528A, designated as R528A) or both sites inactivated (HA1:N285A/HA2/HA3:R528A, designated as N285A/R528A)^7^,^25^,^26^. The N285A complex exhibited dramatically reduced binding to GP2 and weak localization to M cells. The R528A complex also exhibited reduced binding to GP2 and weak localization to M cells, relative to the wild-type HA complex. The double-mutant complex, N285A/R528A, exhibited neither binding to GP2 nor localization to M cells (Fig. 5d,e). Furthermore, the HA complex bound to neither mGP2 (Fig. 5f) nor uromodulin (Supplementary Fig. 5c) expressed in a bacterial expression system yielding completely non-glycosylated proteins. These results indicate that the binding of HA complex to GP2 is mediated by the carbohydrate-binding activities of HA subcomponents, mainly the binding of hexavalent galactose-binding sites of HA1 to galactose moieties in sugar chains present on GP2.

### GP2 serves as a transcytotic receptor for L-PTC *in vivo*

**Figure 6:**
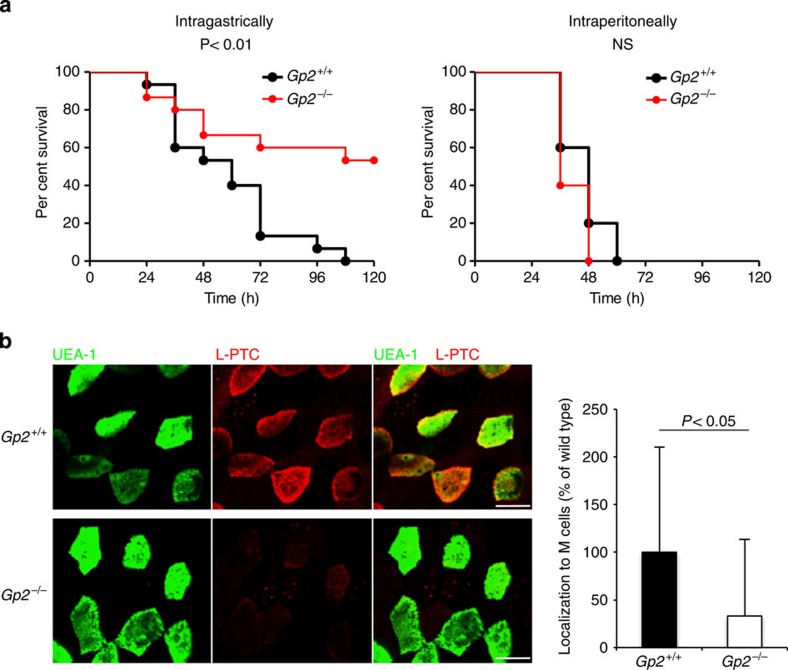
L-PTC is taken up by M cells via the GP2–HA interaction. (**a**) *Gp2*^+/+^ or *Gp2*^−/−^ mice were inoculated intragastrically (1.33 pmol: 1.0 μg) or intraperitoneally (0.067 fmol: 50 pg) with L-PTC (intragastrically*, n*=15 per group; intraperitoneally, *n*=5 per group). Statistical analyses were performed with the log-rank test. (**b**) Alexa Fluor 568-labelled L-PTC (red) were injected into ligated mouse intestinal loops and incubated for 1 h. PPs removed from *Gp2*^+/+^ or *Gp2*^−/−^ mice were incubated with FITC-labelled UEA-1 (green). Images from FAE regions were analyzed quantitatively using the MetaMorph software. The data are expressed as percentages of the level in *Gp2*^+/+^ mice. Error bars indicate s.d. For each group, six different PPs obtained from three different mice were examined (*Gp2*^+/+^, 127 cells; *Gp2*^−/−^, 161 cells). Statistical analyses were performed with Mann–Whitney *U*-test. Scale bar, 10 μm. NS, not significant.

To determine whether GP2 serves as a transcytotic receptor for L-PTC *in vivo*, we used GP2-deficient (*Gp2*^−/−^) mice. These animals exhibited significantly lower susceptibility to orally administered L-PTC than wild-type (*Gp2*^+/+^) mice (Fig. 6a). Consistent with this, uptake of L-PTC by M cells was significantly reduced in *Gp2*^−/−^ mice (Fig. 6b). By contrast, there was no significant difference between *Gp2*^+/+^ and *Gp2*^−/−^ mice in susceptibility to intraperitoneally administered L-PTC (Fig. 6a). Taken together, these results indicate that L-PTC is taken up by M cells via the GP2−HA interaction.

## Discussion

The observations reported here provide evidence that the BoNT/A1 complex exploits M cells to enter the host and exert neurotoxicity. Several studies have reported that experimentally purified BoNT/A1 and its heavy chain domain cross the intestinal epithelial barrier by transcytosis^27^,^28^,^29^,^30^,^31^, and they preferentially recognize a subset of enteroendocrine cells^32^. However, BoNT is present as PTCs associated with NAPs in intestinal fluids^2^. Therefore, the mechanisms underlying the absorption of PTCs are relevant to the pathogenesis of food-borne botulism. We cannot exclude the possibility that L-PTC is also taken up from absorptive epithelium and/or enteroendocrine cells. However, our observation that susceptibility to orally administered L-PTC was dramatically reduced in mice treated with anti-RANKL antibody (that is, M-cell-depleted mice) indicates that M cells are the major site for intestinal translocation of L-PTC.

In this study, we showed that the interaction of HA in the L-PTC with GP2 on the M-cell surface mediates the entry of L-PTC in mice. A similar mechanism may occur in humans, given that GP2 is specifically expressed by human M cells and L-PTC strongly binds human GP2 recombinant protein (Fig. 3a). These observations raise a question: what directs L-PTC to target GP2 on M cells? Indeed, HA binds to various glycoproteins expressed on the apical surface of enterocytes such as mucin 1 (MUC1) and dipeptidyl peptidase-4 (DPP4), as well as those expressed on the surface of lymphoid cells but not M cells^33^: these include programmed cell death-1 (PD-1), T-cell immunoglobulin and mucin protein 3 (TIM3), CD24 and CD40 although the binding to CD40 is weak (Supplementary Fig. 6). Moreover, L-PTC bound to the apical surface of MDCK I monolayers regardless of whether GP2 was present (Fig. 4). These facts suggest that there must be an additional factor involved in the M-cell tropism of HA. Enterocytes are protected from luminal pathogens by the thick layer of mucus and the filamentous brush border glycocalyx (FBBG) on the tip of microvilli, both of which are composed of highly glycosylated mucins^34^. The FAE region has a relatively thin mucus layer compared with other regions of the intestinal mucosa, and this feature tends to promote local contact of antigens and pathogens with the FAE surface^17^. Indeed, our data showed that L-PTC was trapped in the mucus layer and did not reach the enterocytes at the villous epithelium region in *in situ* intestinal loop assays (Supplementary Fig. 7). Furthermore, the FBBG acts as a size-selective barrier to limit the access of particles >30 nm in diameter^35^. Enterocytes in the FAE region have the FBBG, whereas M cells do not. Collectively, these observations suggest that the mucus layer and FBBG that cover enterocytes may exclude L-PTC and HA molecules, which have a diameter of ~32 nm due to the HA moiety^3^, resulting in the M-cell tropism of L-PTC and HA. On M cells, L-PTC mainly recognizes GP2, probably due to its strong affinity for HA and relatively high expression level on M cells.

We showed previously, using an *in vitro* epithelial barrier system, that the interaction of HA with E-cadherin results in disruption of tight junctions, and subsequently to increased uptake of BoNT, primarily by the paracellular route^13^,^14^,^15^. In *in situ* intestinal loop assays in mouse, we observed that the morphology of M cells was disturbed, and fixable fluorescein isothiocyanate (FITC)-dextran 10 K penetrated between M cells and enterocytes after injection of NAPs containing HA (Supplementary Fig. 8). These observations suggest that HA translocated via M cells serves to disrupt the adherens junctions between M cells and enterocytes, thereby compromising the paracellular barrier *in vivo*. Further study is needed to confirm whether this disruption of the paracellular barrier contributes to BoNT absorption *in vivo*.

Uptake of L-PTC from M cells was not abolished completely in *Gp2*^−/−^ mice (Fig. 6b). In this regard, HA engaged in a weak interaction with uromodulin and PrP^C^ in *in vitro* binding assay (Fig. 3a), which could explain the remaining L-PTC uptake by M cells. It should be noted, however, that GP2 was expressed at a much higher level than uromodulin and PrP^C^ (Supplementary Fig. 4). In addition, among these three ligands, L-PTC in the endocytic vesicles co-localized most frequently with GP2. Consistent with these notions, susceptibility to orally administered L-PTC was markedly reduced in *Gp2*^−/−^ mice, suggesting that GP2-mediated endocytosis is the critical pathway for the translocation of L-PTC in the context of food-borne botulism. Further investigation will be needed to elucidate how BoNT deals with the immune surveillance in PPs and disseminates into the systemic circulation. Some BoNT serotypes (for example-, serotypes E and F) cause food-borne intoxication even though they lack genes encoding HA proteins and produce only M-PTCs. Further studies will be required to elucidate the intestinal absorption mechanism of these M-PTCs.

Taken together, our findings shed light on the process of intestinal BoNT invasion, heretofore largely unknown, which is essential. This knowledge could facilitate development of more effective therapies for this disease. Furthermore, techniques for M-cell-targeted delivery of vaccine antigens could greatly improve the efficacy of mucosal vaccines. Thus, exploiting the function of HA represents an attractive approach to development of novel delivery systems for vaccines and drugs^18^,^36^.

## Methods

### Animals

BALB/c mice were purchased from SLC. GP2-deficient mice (*Gp2*^−/−^ mice) were generated as described previously^37^,^38^, and then backcrossed into a BALB/c genetic background. By tissue staining with an anti-GP2 monoclonal antibody, we verified previously that the GP2 knockout abolishes GP2 expression on M cells^21^. These mice were maintained under specific pathogen-free conditions. Female mice were used at the age of 8–12 weeks (Figs 1, 3 and 5 and Supplementary Figs 1,2,4,7 and 8), 8 weeks (Fig. 2 and Supplementary Fig. 3) or 11–19 weeks (Fig. 6). Mice of similar ages were used in comparative analyses. The experimental protocols were approved by the Ethics Review Committee for Animal Experimentation at Osaka University.

### Toxins and non-toxic components

Toxins and non-toxic components were purified from the culture fluid of *C. botulinum* type A strain 62A (ref. 8). Briefly, the organisms were cultured using a cellophane-tube procedure, and the culture supernatant was collected and concentrated by 60% ammonium sulfate precipitation. After dialysis, toxin-containing solution was applied to a SP-Toyopearl 650 M (Tosoh) column. The M-PTC rich fraction was collected and dialysed against 0.01 M sodium phosphate buffer (pH 6.0). L-PTC rich fraction was collected and further purified by lactose gel (EY Laboratories) column, and dialysed against 0.01 M sodium phosphate buffer (pH 6.0). The L-PTC fraction is considered to contain its dimer form, LL-PTC^8^,^9^. The purity of toxins and NAPs were confirmed by SDS–polyacrylamide gel electrophoresis (SDS–PAGE; Supplementary Fig. 9a). Uncropped images of SDS–PAGE gels are shown in Supplementary Fig. 10.

For immunofluorescence staining, toxins were labelled with Alexa Fluor dye using the Alexa Fluor protein labeling kit (Invitrogen). After the reactions, we confirmed the degree of labelling: M-PTC, 7.7 mol dye per mol protein; L-PTC, 18.2 mol dye per mol protein.

### Ligated intestinal loop assay

Mice were not fed for 4 h prior to surgery, but had free access to water. During the procedure, mice were maintained under pentobarbital sodium anaesthesia. Fluorescently labelled toxins (M-PTC and L-PTC, 20 pmol per 100 μl), NAPs (NTNHA/HA, 20 pmol per 100 μl), HA subcomponents (HA1, HA2 or HA3, 60 pmol per 100 μl), reconstituted L-PTC (Alexa Fluor 488-labelled BoNT/Alexa Fluor 568-labelled NAPs, 20 pmol per 100 μl) and reconstituted HAs (Alexa Fluor 568-labelled HA2/HA3 or HA1/Alexa Fluor 568-labelled HA2/HA3, 20 pmol per 100 μl) were injected into ligated intestinal loops containing PPs. After incubation for several hours, mice were euthanized, and PPs were excised from the intestines.

### Immunofluorescence staining

After ligated intestinal loop assays, whole-mount specimens were fixed with 4% paraformaldehyde for 30 min at room temperature, washed with PBS (pH6.0) and then autofluorescence was quenched by incubation in 50 mM NH_4_Cl for 30 min at room temperature. After permeabilization with 0.5% Triton X-100, specimens were incubated with blocking buffer (2% BSA in PBS) for 1 h, followed by incubation with primary antibodies (anti-GP2 (ref. 21) (2.5 μg ml^−1^), anti-uromodulin (10 μg ml^−1^, R&D Systems), anti-PrP^C^ (1.0 μg ml^−1^, SPIBio) and anti-Na^+^K^+^ATPase (2.0 μg ml^−1^, Abcam)) for 2 h at room temperature or overnight at 4 °C. The specimens were then washed with PBS, probed with the Cy3-labelled secondary antibody (3.0 μg ml^−1^, Jackson ImmunoResearch) or Alexa Fluor 488-labelled secondary antibody (10 μg ml^−1^, Invitrogen) for 2 h at room temperature. The specific staining of primary antibodies (anti-GP2, anti-uromodulin and anti-PrP^C^) was confirmed by comparison with an isotype control primary antibodies. In some experiments, specimens were stained for 2 h at room temperature with FITC or rhodamine-labelled UEA-1 (4.0 μg ml^−1^, Vector Laboratories), Alexa Fluor 568-labelled phalloidin (Invitrogen), iFluor 405-labelled phalloidin (Abcam) or Pacific Blue-labelled anti-CD11c antibody (5.0 μg ml^−1^, BioLegend). After immunofluorescence staining, specimens were mounted in ProLong Antifade (Invitrogen) and visualized by confocal microscopy using a CSU21 or CSUX1 confocal scanner unit (Yokogawa) and an IX71 microscope (Olympus). The data were analyzed using the MetaMorph software (Molecular Devices).

For mucin staining, whole-mount specimens were fixed with Carnoy’s solution (60% ethanol, 30% chloroform and 10% glacial acetic acid) for 1 h at room temperature, and then washed with a series of 100% ethanol, 70% ethanol, 40% ethanol and PBS. The specimens were incubated with blocking buffer for 1 h, and then incubated with anti-MUC2 antibody (2.0 μg ml^−1^, Abcam) for 2 h at room temperature. The specimens were washed with PBS, and then probed with Cy3-labelled secondary antibody and iFluor 405-labelled phalloidin for 2 h at room temperature.

For quantitative analysis of localization of L-PTC to M cells, Alexa Fluor 568-labelled L-PTC (20 pmol per 100 μl) was injected into ligated intestinal loops containing PPs and incubated for 1 h. Whole-mount specimens were stained with FITC-labelled UEA-1. Images were taken from FAE and analyzed using the MetaMorph software. The localization value was calculated as follows: (L-PTC area × average intensity of L-PTC in this area)/UEA-1 area.

### Treatment with anti-RANKL antibodies

The anti-mouse RANKL monoclonal antibody (clone IK22-5) was generated by immunizing Sprague Dawley rats with recombinant mouse RANKL and fusing immune lymph node cells with myeloma cells^19^. This antibody was used for *in vivo* RANKL neutralization to transiently deplete M cells^19^,^20^. Mice were injected intraperitoneally with 250 μg of anti-RANKL antibody every 2 days for 8 days. As a negative control, another group of mice was treated with a purified functional-grade isotype control rat immunoglobulin-G2a (IgG2a) antibody (eBioscience).

### Comparison of toxicity

Mice were not fed for 4 h prior to administration, but had free access to water. Mice were inoculated intragastrically by gavage with 0.3 ml of 10 mM sodium phosphate buffer (pH 6.0)+0.1% gelatin containing various concentrations of toxins. Alternatively, mice were injected intraperitoneally with 0.3 ml of 10 mM sodium phosphate buffer (pH 6.0)+0.1% gelatin containing various concentrations of toxins. We used a toxin dose at which almost all control mice presented with flaccid quadriplegia ~24 h after the administration of PTCs. Mice were observed for morbidity, mortality and survival time over the course of 3 weeks. The investigators were ‘blinded’ to group allocation both during the experiment and when assessing the outcome (Figs 2b, 6a and Supplementary Fig. 3).

### Preparation of HA proteins

Purified DNA from *C. botulinum* type A strain 62A was used as a template for the amplification of DNA encoding HA1, 2 and 3 by PCR. The amplified DNAs were inserted into pET-52b(+) (Merck Millipore) and pT7-FLAG-1 (Sigma-Aldrich). Recombinant HAs were expressed as N-terminal Strep (Strep–HA1, Strep–HA3) or FLAG-tagged (FLAG–HA2) or C-terminal FLAG-tagged (HA1–FLAG) proteins in *E. coli* strain Rosetta2 (DE3) (Merck Millipore) or BL21-CodonPlus (DE3)-RIL (Agilent Technologies), and purified using StrepTrap HP (GE Healthcare) or anti-FLAG M2 resin (Sigma-Aldrich), respectively. Carbohydrate-binding mutants of HA1 and HA3 were obtained by site-directed mutagenesis using the PrimeSTAR Max polymerase (TAKARA BIO)^26^. Briefly, Asn285 in HA1 and Arg528 in HA3, were substituted with alanine (HA1N285A and HA3R528A)^7^,^25^. The purity of HA proteins were confirmed by SDS–PAGE (Supplementary Fig. 9a). Primer sets are listed in Supplementary Table 1. For reconstitution of the HA complex, recombinant HA1, HA2, and HA3 proteins were mixed at a molar ratio of 4:4:1 (20 μM:20 μM:5 μM) in PBS (pH 7.4) and incubated for 3 h at 37 °C. Reconstitution of HA complex was confirmed by pull-down using Strep-Tactin Superflow agarose (Novagen) and SDS–PAGE (Supplementary Fig. 9b,c). The molarities of HA1, HA2 and HA3 are expressed as the concentrations of their respective monomeric forms. The molarity of the HA complex is expressed as the concentration of HA3 in the trimeric form.

For immunofluorescence staining, HA proteins were labelled with Alexa Fluor dye. The degrees of labelling: Strep–HA1, 1.4 mol dye per mol protein; FLAG–HA2, 0.9 mol dye per mol protein; Strep–HA3, 2.1 mol dye per mol protein.

### Preparation of Fc-fusion proteins

To obtain expression vector for fusion proteins of mouse GP2, human GP2, mouse uromodulin (Umod) or mouse PrP^C^ with the Fc segment of human IgG1 (mGP2-Fc, hGP2-Fc, Umod-Fc and PrP^C^-Fc), complementary DNAs (cDNAs) corresponding to the extracellular domains of each protein were amplified by PCR using primer sets listed in Supplementary Table 1. The cDNA fragments were inserted into the pcDNA3 expression vector (Invitrogen) containing a fragment encoding the Fc segment of human IgG1 to generate Fc-fusion proteins^21^,^22^. Human embryonic kidney 293T (HEK293T) cells were transiently transfected with the expression vectors using Lipofectamine 2000 (Invitrogen), and then cultured for several days. The Fc-fusion proteins secreted into the culture supernatant were purified on HiTrap protein A HP columns (GE Healthcare).

### HA pull-down assay

Recombinant mouse GP2-Fc, human GP2-Fc, mouse uromodulin-Fc, mouse PrP^C^-Fc, human MUC1-Fc (Life Technologies), human DPP4-Fc (Life Technologies), mouse PD-1-Fc (R&D Systems), mouse TIM3-Fc (R&D Systems), human CD40-Fc (Enzo), human CD24-Fc (Life Technologies) or control Fc protein at a final concentration of 5.0 μg ml^−1^ was incubated for 2 h at 4 °C with Strep-Tactin Superflow agarose pre-bound with HA (HA subcomponent or a complex of HA1, HA2 and HA3). After washing with PBS-0.05% Triton X-100, HA and HA-bound proteins were eluted from the resin with SDS–PAGE sample buffer and analyzed by immunoblotting using horseradish peroxidase (HRP)-labelled anti-human IgG antibody (diluted 1:10^4^, Jackson ImmunoResearch). Uncropped images of immunoblots are shown in Supplementary Fig. 10.

### Preparation of GST-fusion proteins

Recombinant mouse GP2 protein lacking the transmembrane domain and mouse uromodulin protein lacking the transmembrane domain were expressed in *E. coli* strain Rosetta2 (DE3) or BL21-CodonPlus (DE3)-RIL as N-terminal glutathione S-transferase (GST)-fusion proteins. Primer sets are listed in Supplementary Table 1. These proteins were purified with a GSTrap column (GE Healthcare) and used for pull-down assays. GST-fusion proteins were detected by immunoblotting using an anti-GST antibody (diluted 1:5,000, BioAcademia) and HRP-labelled anti-rabbit IgG antibody (diluted 1:10^4^).

### *In situ* paracellular flux assay

Mice were not fed for 4 h prior to surgery, but had free access to water. During the procedure, mice were maintained under pentobarbital sodium anaesthesia. NAPs (NTNHA/HA, 500 pmol) and paracellular tracer (fixable FITC-dextran 10 K; D-10, 2.0 mg ml^−1^, Invitrogen, 100 μl) or vehicle control (PBS) and the tracer were injected into ligated mouse intestinal loops and incubated for 2 h, and FAE regions were stained with rhodamine-labelled UEA-1. After immunofluorescence staining, the morphology of M cells and localization of paracellular tracer were observed by confocal microscopy.

### Quantitative reverse-transcription PCR

Total RNA was isolated from FAE of BALB/c mouse using the TRIzol (Life Technologies) and subjected to reverse transcription using the PrimeScript RT reagent kit (TAKARA BIO). The cDNA samples were amplified with the Eco Real Time PCR System (Illumina), SYBR premix Ex Taq (TAKARA BIO) and the primer sets specific for mouse genes. Primer sets are listed in Supplementary Table 1.

### Enzyme-linked immunosorbent assay

Binding of L-PTC to recombinant mouse GP2-Fc was analyzed by enzyme-linked immunosorbent assay (ELISA). Plates were coated with recombinant mouse GP2-Fc protein at a final concentration of 2.5 μg ml^−1^ in PBS (pH 7.4) overnight at 4 °C. Wells were washed three times with PBS (pH 6.0), blocked with 1% BSA in PBS and incubated with L-PTC for 1 h at room temperature. After washing, rabbit anti-BoNT polyclonal antibody (diluted 1:3,000) was added and plates were incubated for 1 h at room temperature. After washing, HRP-labelled anti-rabbit IgG antibody (diluted 1:10^4^) was added, and the sample was incubated for an additional 1 h at room temperature. Plates were then treated with ABTS (Roche) and absorbance values (optical density: 405 nm) were measured. In the competition assay, L-PTC was pre-incubated with sugar chain for 1 h at room temperature prior to ELISA.

### Glycopeptidase F treatment

Recombinant mouse GP2-Fc was treated with Glycopeptidase F (PNGase F, TAKARA BIO) to remove N-linked glycan from GP2. Briefly, 3 μg of mGP2-Fc was incubated with Glycopeptidase F (5.0 mU) for 24 h at 37 °C under native (detergent free) conditions. Deglycosylation of mGP2-Fc was confirmed by SDS–PAGE, and the deglycosylated protein was used for ELISA.

### Immunofluorescence staining in GP2-expressing MDCK I cells

MDCK I cells (European Collection of Cell Cultures) were cultured in minimum essential medium (Gibco) supplemented with 1.0 mM L-glutamine and antibiotics (penicillin G, 70 U ml^−1^; streptomycin, 70 μg ml^−1^), 10% heat-inactivated foetal bovine serum, 17.86 mM NaHCO_3_ (Wako) and 15 mM HEPES (Dojindo) at pH 7.4 (ref. 14). Cells were transiently transfected with vector encoding mouse GP2 using ScreenFect A (Wako) and seeded on Transwell pore filter (Costar). After 3 days, cells were apically treated with L-PTC. After various times, cells were fixed with 4% paraformaldehyde for 20 min and permeabilized with 0.5% Triton X-100 for 5 min, and then incubated with blocking buffer (5% BSA in PBS) for 1 h. The cells were incubated with anti-L-PTC (diluted 1:400) and anti-GP2 antibodies (1.0 μg ml^−1^) and then probed with Alexa Fluor 488- (5.0 μg ml^−1^) or Cy3-labelled secondary antibodies (1.5 μg ml^−1^).

### Statistical analyses

Student’s *t*-test was used to compare the binding of L-PTC with Fc-fusion proteins; the co-localization of L-PTC with GP2, uromodulin or PrP^C^ in M cells and the endocytotic vesicles of L-PTC in MDCK I cells. The Mann–Whitney *U*-test was used to compare the localization of L-PTC to M cells in FAE. The Kaplan–Meier estimator method was used to draw survival curves, and a log-rank test was used to compare the survival rates. *P* values<0.05 were considered to be statistically significant.

## Author contributions

T.M., H.O., and Y.F. designed the research and analyzed the data. T.M. performed the majority of the experiments and the analyses. Y.S., M.Y., and S.A. provided recombinant materials and analyzed the data. H.Y. provided the anti-RANKL antibody. T.K. provided toxins and anti-BoNT antibodies. S.F. provided GP2-deficient mice. Y.N. performed the qPCR assays and analyzed the data. K.H., S.F. and H.O. provided Fc-fusion proteins. T.M., H.O., K.H. and Y.F. co-wrote the manuscript. All authors participated in the discussion of the data and in production of the final version of the manuscript.

## Additional information

**How to cite this article**: Matsumura, T. *et al.* Botulinum toxin A complex exploits intestinal M cells to enter the host and exert neurotoxicity. *Nat. Commun.* 6:6255 doi: 10.1038/ncomms7255 (2015).

## Supplementary Material

Supplementary Figures and Supplementary Table Supplementary Figures 1-10 and Supplementary Table 1

Supplementary Movie 1 Co-localization of reconstituted L-PTC and dendritic cells (DCs). Transport of reconstituted L-PTC from an M cell to an underlying DC (see Supplementary Fig. 1 for the full legend).

Supplementary Movie 2 Co-localization of L-PTC and GP2. Co-localization of Alexa Fluor 568-labeled L-PTC and GP2 (detected with anti-GP2 antibody and Alexa Fluor 488-labelled anti-rat IgG) in M cells.
