# Supplementary material for: Botulinum toxin A complex exploits intestinal M cells to enter the host and exert neurotoxicity
Source: Nat Commun. 2015 Feb 17;6:6255. doi: 10.1038/ncomms7255 (PMC4339894; doi:10.1038/ncomms7255)
Supplement: Supplementary Figures and Supplementary Table — Supplementary Figures 1-10 and Supplementary Table 1 [file ncomms7255-s1.pdf]

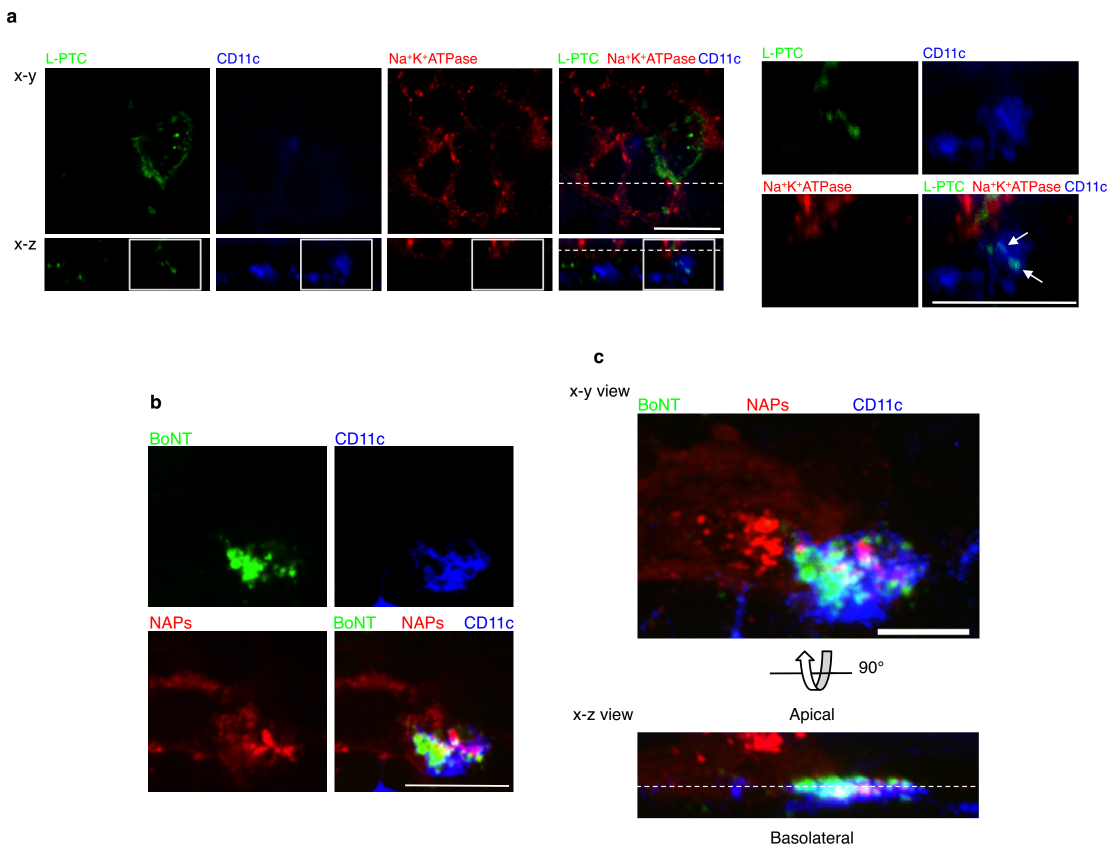

Supplementary Figure 1: Co-localization of reconstituted L-PTC and dendritic cells (DCs). (**a**) Alexa Fluor 488–labeled L-PTC (green) was injected into ligated mouse intestinal loops and incubated for 3 h; FAE regions were stained with a anti-$Na^{+}K^{+}$ATPase antibody (basolateral marker), Cy3–labeled anti-rabbit IgG antibody (red), and Pacific Blue–labeled anti-CD11c antibody (DC marker, blue). X-Z images in lower panels correspond to the positions indicated by dotted lines in the X-Y images. Right panels show a higher-magnification image of the boxed region. L-PTC were captured by DCs in the SED (arrows). (**b** and **c**) For reconstitution of the L-PTC, Alexa Fluor 488–labeled BoNT (green) and Alexa Fluor 568–labeled NAPs (red) were mixed at a molar ratio of 5:1 (1000 nM : 200 nM) in PBS (pH 6.0), and then incubated for 3 h at 37°C. Reconstituted L-PTC was confirmed by pull-down using lactose gel beads (EY Laboratories, Inc.),

which bound to HA, followed by immunoblotting with anti-L-PTC antibody (data not shown). Reconstituted L-PTC was injected into ligated intestinal loops and incubated for 3 h. FAE was stained with a Pacific Blue–labeled anti-CD11c antibody (blue). (**b**) X-Y view. (**c**) Three-dimensional data, Top, X-Y view. Bottom, side (X-Z) view. X-Y images (**b**) correspond to the position indicated by dotted line in the X-Z view (**c**). For three-dimensional imaging, see Supplementary Movie 1. Scale bars: 10 μm (**a, b,** and higher-magnification images in **a**); 5 μm (**c**). Data are representative of two independent experiments.

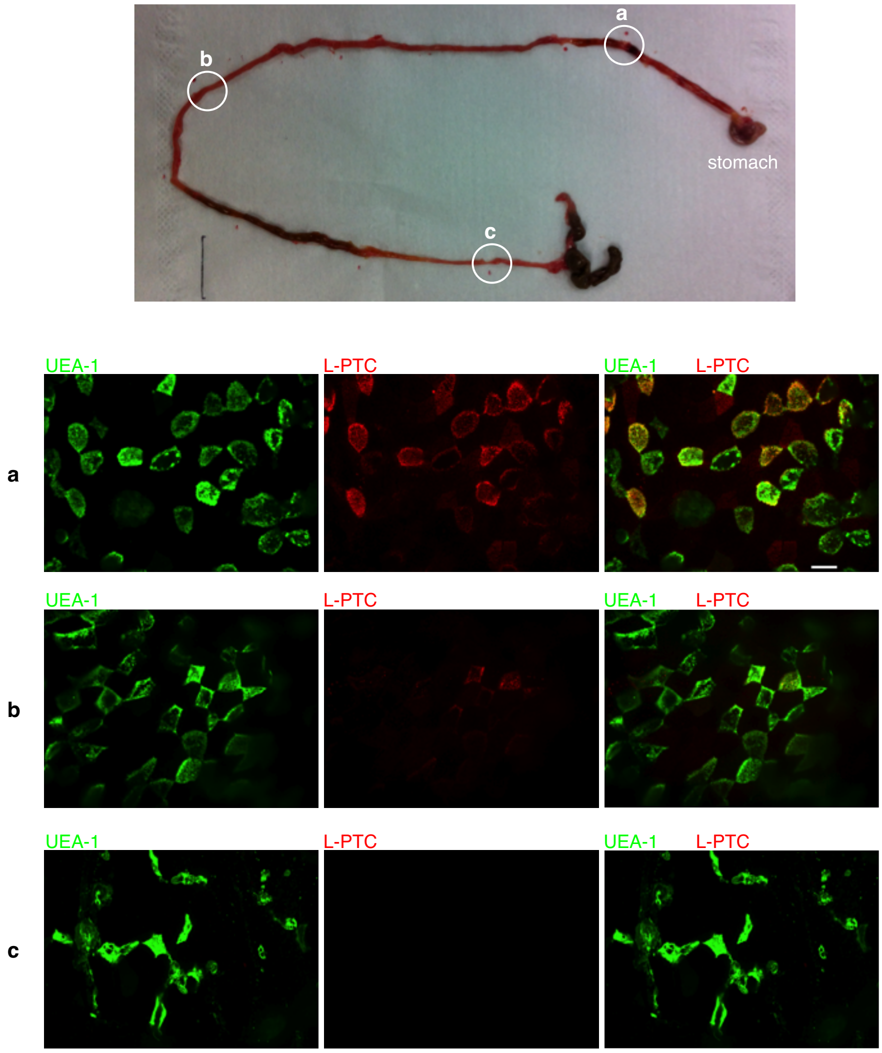

Supplementary Figure 2: Localization of orally administered L-PTC in small intestine. Alexa Fluor 568–labeled L-PTC (26.7 pmol: 20 µg, red) was intragastrically administered to mice and incubated for 1 h. After incubation, the small intestine was removed, and PPs were excised from this tissue. PPs from duodenum (**a**), jejunum (**b**), or ileum (**c**) were stained with FITC–labeled UEA-1 (green), and analyzed on a confocal laser microscope. Scale bar, 10 µm. Data are representative of two independent experiments.

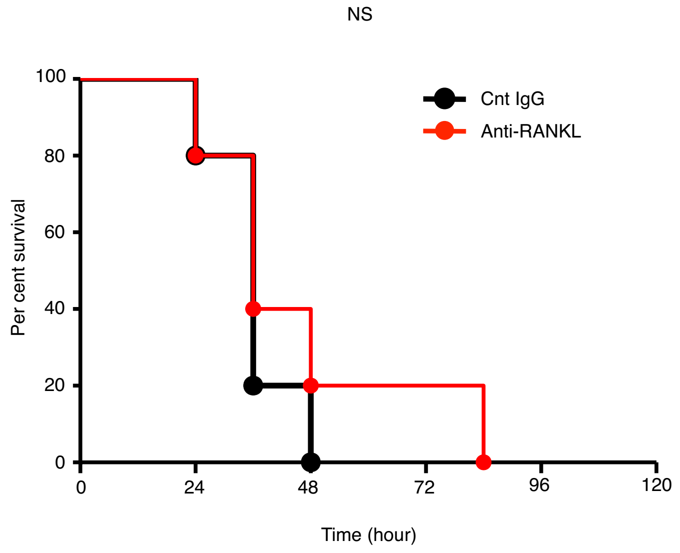

Supplementary Figure 3: M-cell depletion does not influence lethality upon oral administration of M-PTC. Mice were treated intraperitoneally with anti-RANKL antibody or control rat IgG. M-PTC were intragastrically (174.4 pmol: 50 µg) administered to mice treated with anti-RANKL antibody or rat IgG (n = 5 per group). Statistical analyses were performed with the log-rank test. NS, not significant.

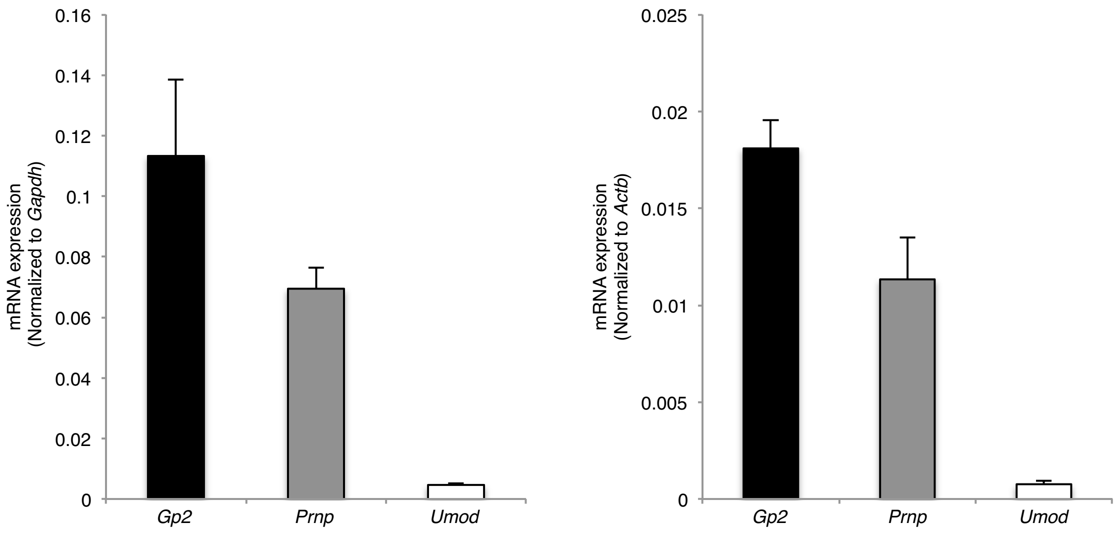

Supplementary Figure 4: qPCR analysis of mouse GP2, PrP^{C}, and uromodulin mRNAs. Total RNA was prepared from FAE. Expression of each gene was analyzed by real-time qPCR. The expression levels of mRNAs encoding GP2 (*Gp2*), PrP^{C} (*Prnp*), and uromodulin (*Umod*) were normalized to the level of the mRNA encoding glyceraldehyde-3-phosphate dehydrogenase gene (*Gapdh*, **a**) or β-actin (*Actb*, **b**). Error bars indicate s.d. (*n* =3).

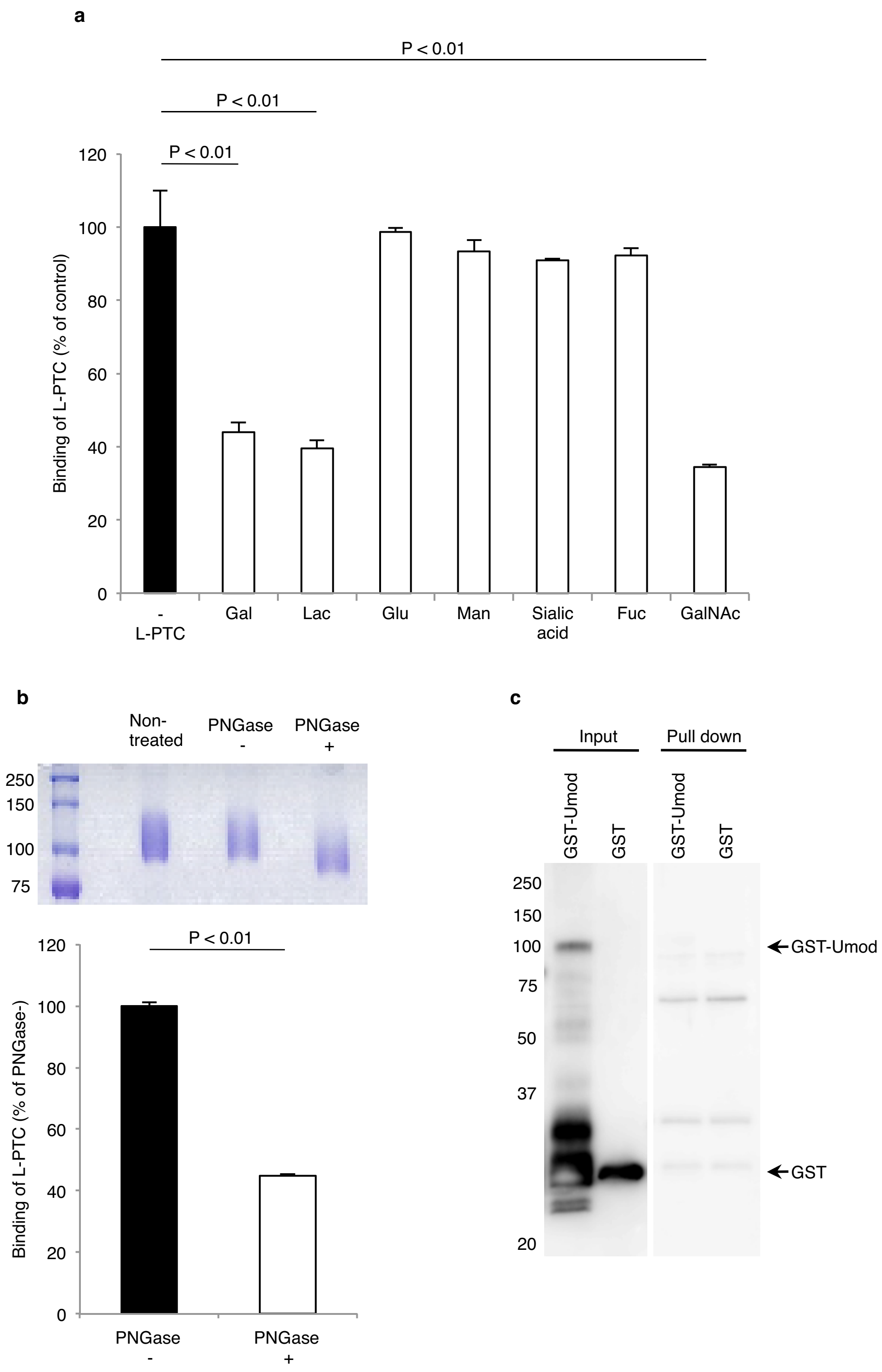

Supplementary Figure 5: Binding of L-PTC to GP2 and uromodulin is mediated by sugar chains. (**a**) L-PTC (100 nM) was pre-incubated with galactose (Gal), lactose (Lac),

glucose (Glu), mannose (Man), sialic acid, fucose (Fuc), or N-acetylgalactosamine (GalNAc) (10 mM), and then added to plates coated with mGP2-Fc proteins. Bound L-PTC was detected using anti-BoNT and HRP–labeled anti-rabbit IgG antibodies. The data are expressed as a percentage of the binding of L-PTC in the absence of added carbohydrates. Error bars indicate s.d. (n = 3). Statistical analyses were performed with the Student's *t*-test. (**b**) Deglycosylation of mGP2-Fc was confirmed by SDS-PAGE. Binding of L-PTC to deglycosylated mGP2-Fc was analyzed by ELISA. Bound L-PTC was detected using anti-BoNT and HRP–labeled anti-rabbit IgG antibodies. Error bars indicate s.d. (n = 3). Statistical analyses were performed with the Student's *t*-test. (**c**) GST-mouse uromodulin or GST was incubated with Strep-Tactin Superflow agarose pre-bound to HA. HA-bound proteins were analyzed by immunoblotting with anti-GST antibody and HRP–labeled anti-rabbit IgG antibody.

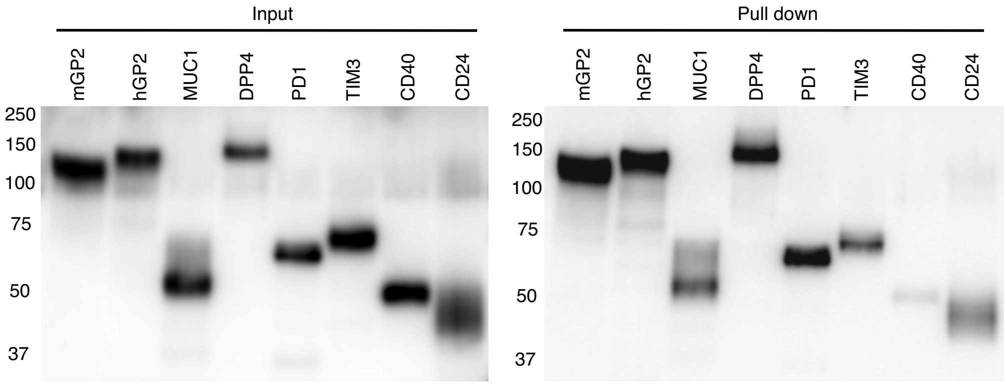

Supplementary Figure 6: HA binds to various glycoproteins. Recombinant Fc proteins were incubated with Strep-Tactin Superflow agarose pre-bound to HA. HA-bound proteins were analyzed by immunoblotting using HRP–labeled anti-human IgG antibody. Data are representative of two independent experiments.

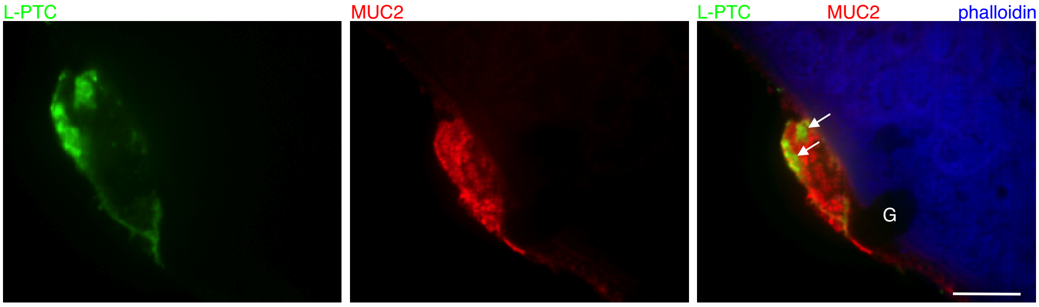

Supplementary Figure 7: L-PTC is trapped into the mucus layer. Alexa Fluor 488–labeled L-PTC (green) were injected into ligated mouse intestinal loops and incubated for 1 h. Whole-mount specimens were fixed with Carnoy's solution and stained with anti-MUC2 antibody and Cy3–labeled anti-mouse IgG antibody (red) and iFluor 405–labeled phalloidin (blue). L-PTC was trapped in the mucus layer (arrows). G: goblet cell. Scale bar, 10 µm. Data are representative of two independent experiments.

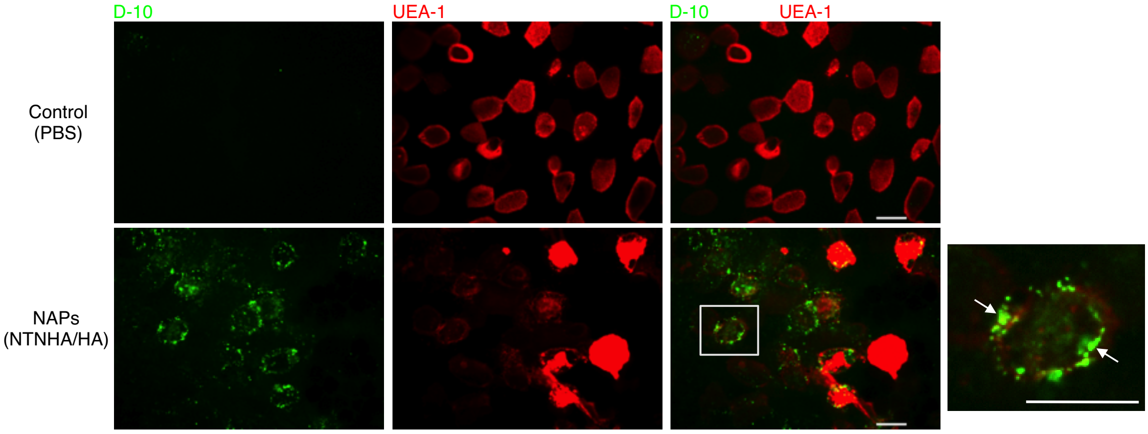

Supplementary Figure 8: NAPs (NTNHA/HA) disrupt the paracellular barrier around the M cells. NAPs (NTNHA/HA) and paracellular tracer (fixable FITC–dextran 10K; D-10, green) or vehicle control (PBS) and the tracer were injected into ligated mouse intestinal loops and incubated for 2 h, and FAE regions were stained with rhodamine-labeled UEA-1 (red). Right panel shows a higher-magnification image of the boxed region. Paracellular tracer penetrated between M cells and enterocytes (arrows). Scale bars: 10 μm. Data are representative of two independent experiments.

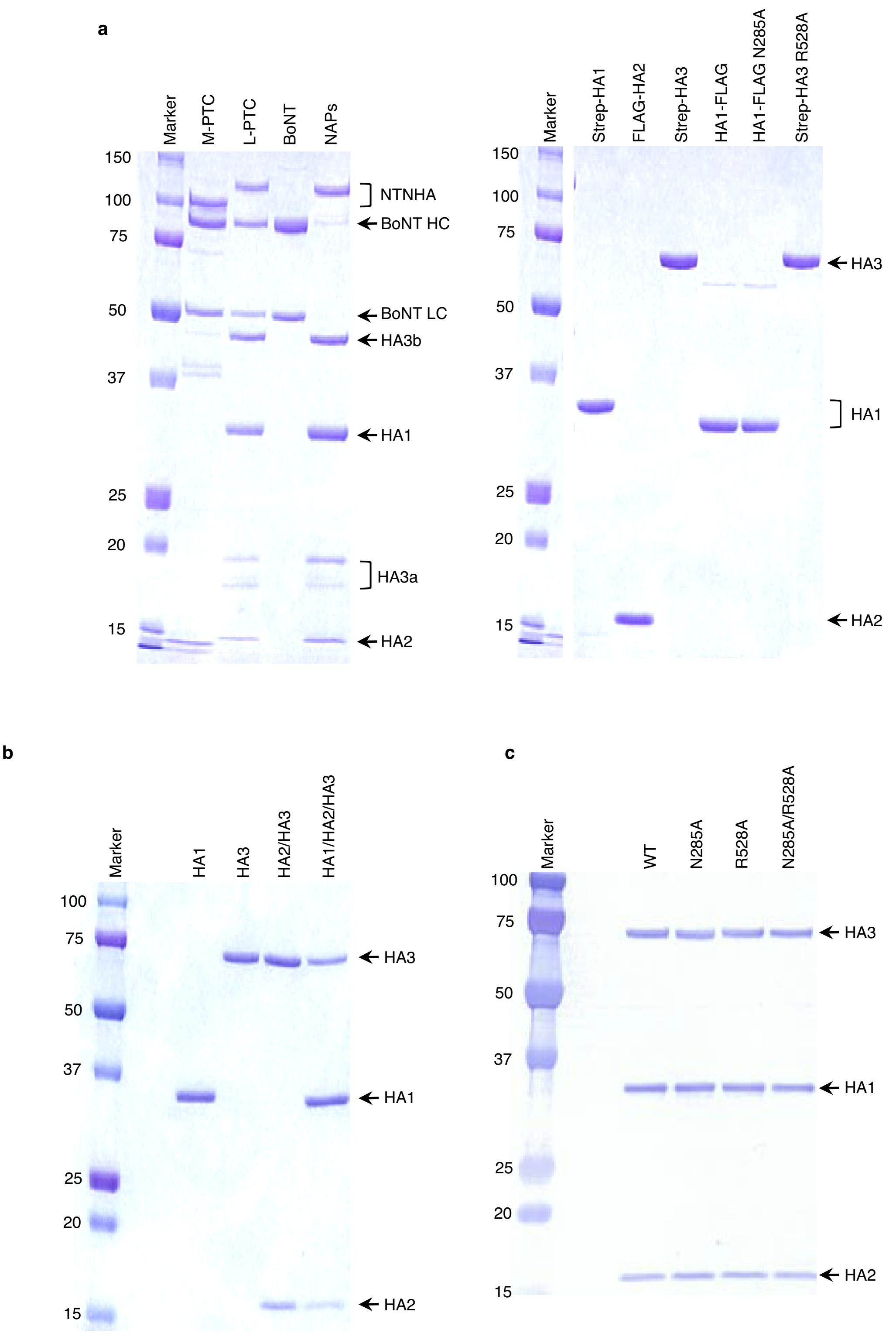

Supplementary Figure 9: Toxins, NAPs, and recombinant HA proteins used in this

study. (**a**) SDS-PAGE of M-PTC, L-PTC, BoNT, NAPs (2.0 μg), and recombinant HA protein (1.0 μg). HA3 protein is cleaved to yield two fragments, designated HA3a and HA3b (alternatively termed HA20 and HA52, respectively), in L-PTC and NAPs[8]. (**b** and **c**) Recombinant HA1, HA2, and HA3 proteins were mixed at a molar ratio of 4:4:1 (20 μM:20 μM:5 μM) in PBS (pH 7.4), and then incubated for 3 h at 37°C. Reconstituted HA (complex of Strep-HA1, FLAG-HA2, and Strep-HA3, **b**) and reconstituted HA harboring mutant HA1 or HA3 (complex of HA1-FLAG [N285A], FLAG-HA2, and Strep-HA3 [R528A], **c**) were confirmed by pull-down using Strep-Tactin Superflow agarose and SDS-PAGE.

Fig. 3a

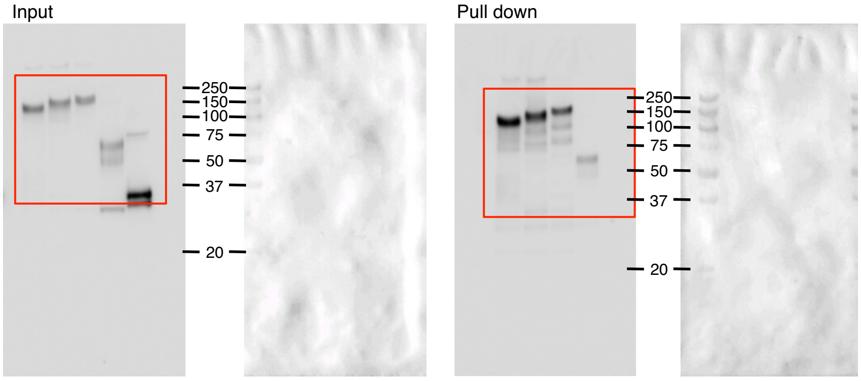

Fig. 5c

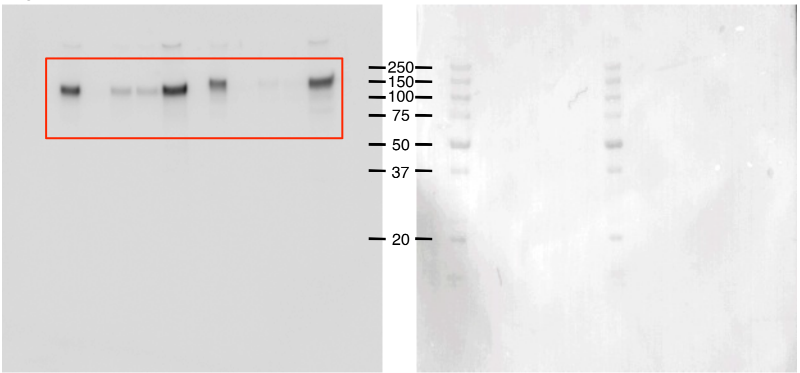

Fig. 5e

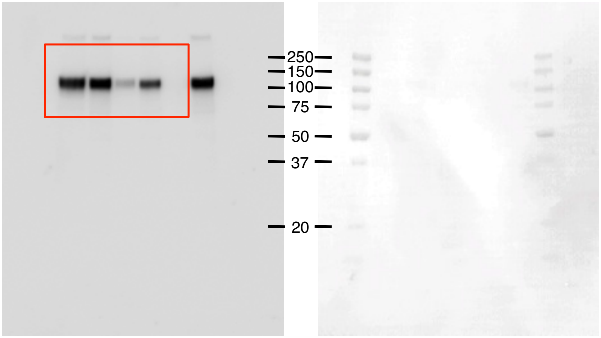

Fig. 5f

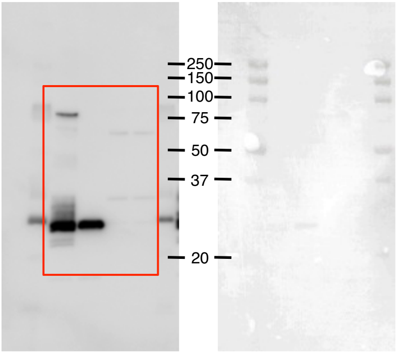

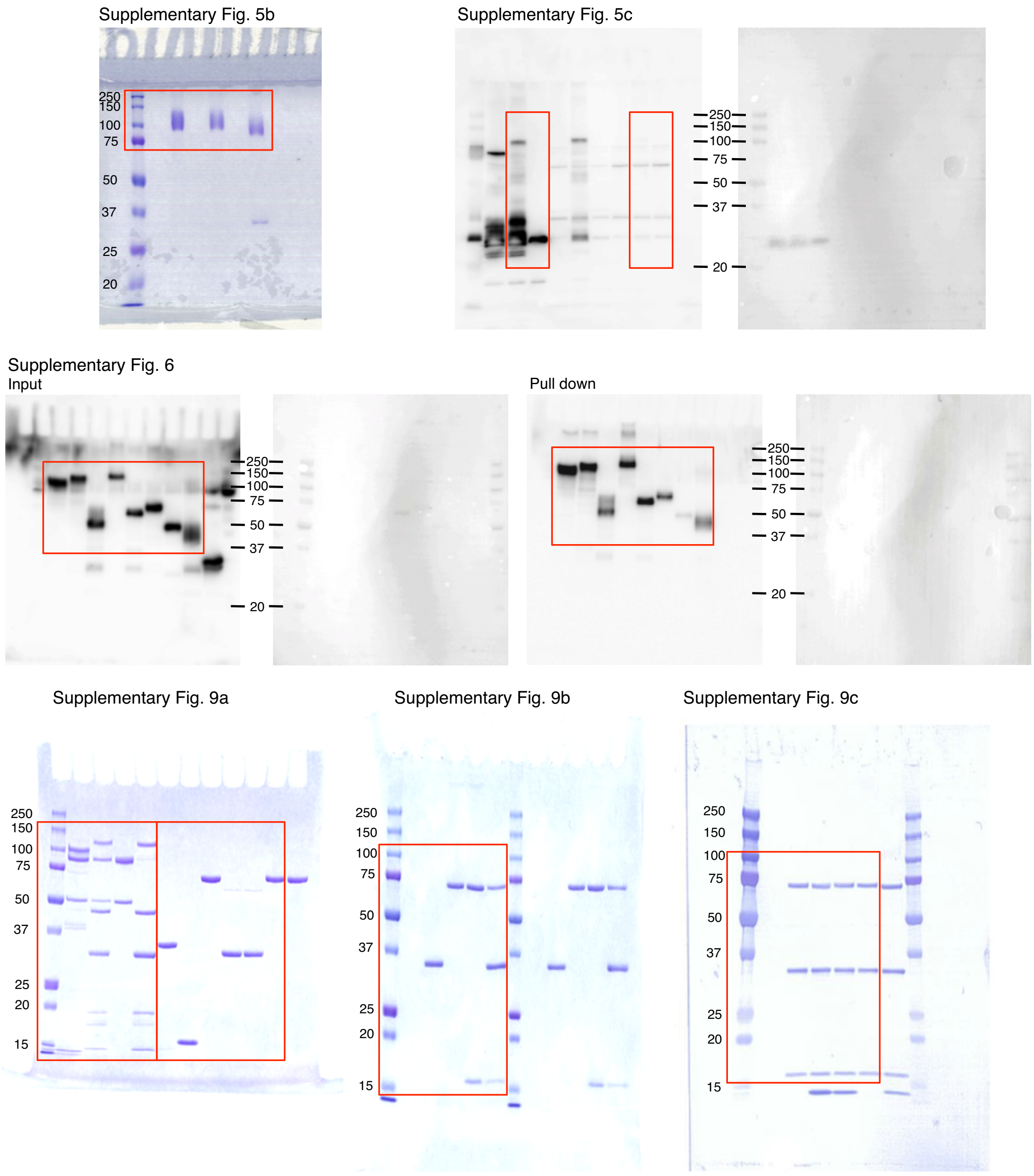

Supplementary Figure 10: Uncropped scan images of immunoblots and SDS-PAGE gels (CBB staining). The red boxed regions in the uncropped scan images were used in the manuscript. The right side of the uncropped chemiluminescence image is a bright-field image of the blot membrane, showing molecular markers (kDa).

Supplementary Table 1: Primers sequences used in this study.

Primer	sequence (5'-3')
Strep-HA1-F	GGAACGGTACCCAGTAATCCAAAATTCATTAAATG
Strep-HA1-R	ATAGTCGACTTATGGGTTACGAATATTCCA
FLAG-HA2-F	TGAATAAGCTTTCAGTTGAAAGAACTTTTCTAC
FLAG-HA2-R	CTTTGGTACCTTATATTTTTTCAAGTTTGAAC
Strep-HA3-F	AAAGTTAGGTACCCTAGTGATACTATTGATTTAG
Strep-HA3-R	CGTGTCGACTTAATTAGTAATATCTATATGC
HA1-FLAG-F	CATGCCATGGTAATCCAAAATTCATTAAA
HA1-FLAG-R	CGGGATCCTTACTTGTCATCGTCATCCTTGTAGTCTGGGTTACGAATATTCCATTTC
HA1-FLAG N285A-F	GATGATGCTCAGAAATGGAATATTCGT
HA1-FLAG N285A-R	TTTCTGAGCATCATCTCCATGATAATT
Strep-HA3 R528A-F	TATACAGCTCAAAGCCCTGATGTCCATG
Strep-HA3 R528A-R	GCTTTGAGCTGTATAGTAATGTGCACC
mGP2-Fc-F	GAAGATCTACCATGAAAAGGATGGTGGGTT
mGP2-Fc-R	CCGCTCGAGTGTAGTGTGGGGAGTCCCC
hGP2-Fc-F	CGCGGATCCACCATGGAAAGGATGGTGGGC
hGP2-Fc-R	CCGCTCGAGTCCATTCATGACACCGGGAGA
Umod-Fc-F	CGCAGATCTACCATGGGGATCCCTTTGACC
Umod-Fc-R	CGCGTCGACCTTGGACACTGAGGCCTGG
PrP^{C}-Fc-F	CGGGATCCACCATGGCGAACCTTGGCTACT

Supplementary Table 1: Primers sequences used in this study (continued).

Primer	sequence (5'-3')
PrP^C-Fc-R	CGCTCGAGGGATCTTCTCCCGTCGTAATAG
GST-mGP2-F	TCCAGGGGCCCCTGGGATCTACAATACATCAAGGTTATGGGAG
GST-mGP2-R	ATGCGGCCGCTCGAGTCATGTAGTGTGGGGAGTCCCCTTC
GST-Umod-F	TCCAGGGGCCCCTGGGAAGTAACTCAACAGAAGCGAGAC
GST-Umod-R	ATGCGGCCGCTCGAGTCACTTGGACACTGAGGCCTGGAC
Used for qPCR	
Actb-F	GAGCGCAAGTACTCTGTGTG
Actb-R	AACGCAGCTCAGTAACAGTCC
Gapdh-F	TGTGTGCGTCGTGGATCTGA
Gapdh-R	TTGCTGTTGAAGTCGCAGGAG
Gp2-F	AACTGCTATGCCACCCCTTC
Gp2-R	TCGGCTTTCTGAGGACACAC
Umod-F	CAGGTGTTTCCAGGACAGAGG
Umod-R	CATTCAGAACACCGTCTCGC
Prnp-F	GGTCCCTTTGATGGAGTCTGTC
Prnp-R	TGTGGATGCTCTAGCTATCCCA
Used for mGP2 transfection	
mGP2-F	GAAGATCTACCATGAAAAGGATGGTGGGTT
mGP2-R	CCGCTCGAGGAACAGTAGAGCCAGGAAGAC
